# Co-actors Exhibit Similarity in Their Structure of Behavioural Variation That Remains Stable Across Range of Naturalistic Activities

**DOI:** 10.1038/s41598-020-63056-x

**Published:** 2020-04-14

**Authors:** Lillian M. Rigoli, Tamara Lorenz, Charles Coey, Rachel Kallen, Scott Jordan, Michael J. Richardson

**Affiliations:** 1 Department of Psychology, Macquarie University, Sydney, New South Wales Australia; 2 Center for Cognition, Action & Perception, Department of Psychology, University of Cincinnati, Cincinnati, OH USA; 3 Department of Mechanical and Materials Engineering, University of Cincinnati, Cincinnati, OH USA; 4 Department of Electrical Engineering, University of Cincinnati, Cincinnati, OH USA; 5 Osher Center for Integrative Medicine, Harvard Medical School and Brigham and Women’s Hospital, Boston, MA USA; 6 Centre for Elite Performance, Expertise and Training, Macquarie University, Sydney, New South Wales Australia; 7 Department of Psychology, University of Illinois, IL, USA

**Keywords:** Psychophysics, Human behaviour

## Abstract

Human behaviour, along with any natural/biological behaviour, has varying degrees of intrinsic ‘noise’ or variability. Many studies have shown that the structure or patterning of this variability is sensitive to changes in task and constraint. Furthermore, two or more humans interacting together often begin to exhibit similar structures of behavioural variability (i.e., the patterning of their behavioural fluctuations becomes aligned or matched) independent of any moment-to-moment synchronization (termed *complexity matching*). However, much of the previous work has focused on a subset of simple or contrived behaviours within the context of highly controlled laboratory tasks. In the current study, individuals and pairs performed five self-paced (unsupervised), semi-structured activities around a university campus. Empatica E4 wristbands and iPhones were used to record the participants’ behavioural activity via accelerometers and GPS. The results revealed that the structure of variability in naturalistic human behaviour co-varies with the task-goal constraints, and that the patterning of the behavioural fluctuations exhibited by co-acting individuals does become aligned during the performance of everyday activities. The results also revealed that the degree of complexity matching that occurred between pairs remained invariant across activity type, indicating that this measure could be employed as a robust, task-independent index of interpersonal behaviour.

## Introduction

When measuring any human movement or behaviour over time, the resulting measurements always contain various magnitudes of natural variability. This is true whether one considers an individual’s reaction times to repeated environmental stimuli^1^, the spatial accuracy of an individual when hitting a nail with a hammer^2,3^, or an individual’s stride length when walking or running^4,5^. Even an individual’s head and postural position while sitting or standing still fluctuates over time^6^. Traditionally, such behavioural variation was assumed to be random or non-functional sensorimotor or measurement ‘noise’^7–9^. However, there is now a growing body of research demonstrating that the behavioural fluctuations that occur during natural human activity are typically correlated over time (i.e., exhibit varying degrees of self-similarity and non-randomness)^1,10–13^.

Of particular relevance here, is that research exploring the patterning of human behavioural variability has demonstrated how the structure of such variation is sensitive to changes in task and environmental constraint, with some patterns of behavioural variance being more or less random than others^11,12,14^. Recent research suggests that the patterning of co-actors’ behavioural variability can also become aligned or ‘matched’ together during task performance, even when the movements or actions of the co-actors are not locally synchronized or coordinated. This latter phenomenon has been termed *complexity matching* and implies that the behavioural actions and movements of co-acting individuals can become entrained at a more global level during social interaction. However, the majority of research exploring the structure of human behavioural variability and complexity matching among co-actors has been conducted using highly constrained laboratory-based tasks, and it remains unclear whether these findings generalize to activities performed outside the laboratory. Accordingly, the current study aimed to validate these findings within the context of everyday, real-world activities.

## The Structure of Behavioural Variability

**Figure 1:**
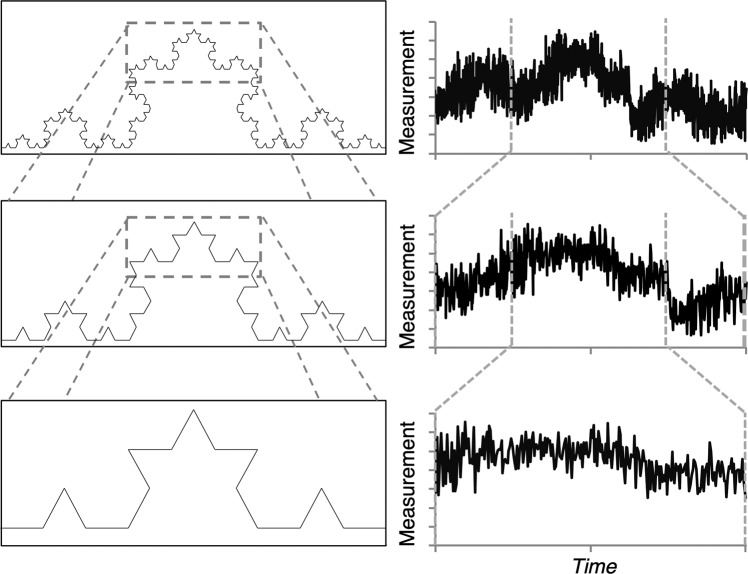
(Left) The Koch curve at three levels of magnification. (Right) A time series of behavioural measurements at three levels of magnification. In both cases, the patterns observed at every level of magnification are self-similar (see text for more details). Adapted from^15^.

The term fractal is commonly used to refer to geometric objects or structures like the Koch curve illustrated in the left panel of Fig. 1. The Koch curve, like all geometric fractals, is simply a recursive structure – a structure where a pattern is repeated at decreasing (or increasing) scales to form a whole – such that a fractal object is simply an object that has the same or *self-similar structure at any scale of observation*.

The variability present in human behaviour can also exhibit fractal-like properties in that such variability is often *statistically self-similar* with respect to time^16^. More specifically, when one measures human behaviour over time (i.e., takes sequential measurements of a behaviour or behavioural event), the fluctuations in the resulting behavioural time series typically contain long-range correlations or nested patterns of variability. This is illustrated in the right panel of Fig. 1, where the variance or fluctuations observed across large and small timescales is statistically similar.

**Figure 2:**
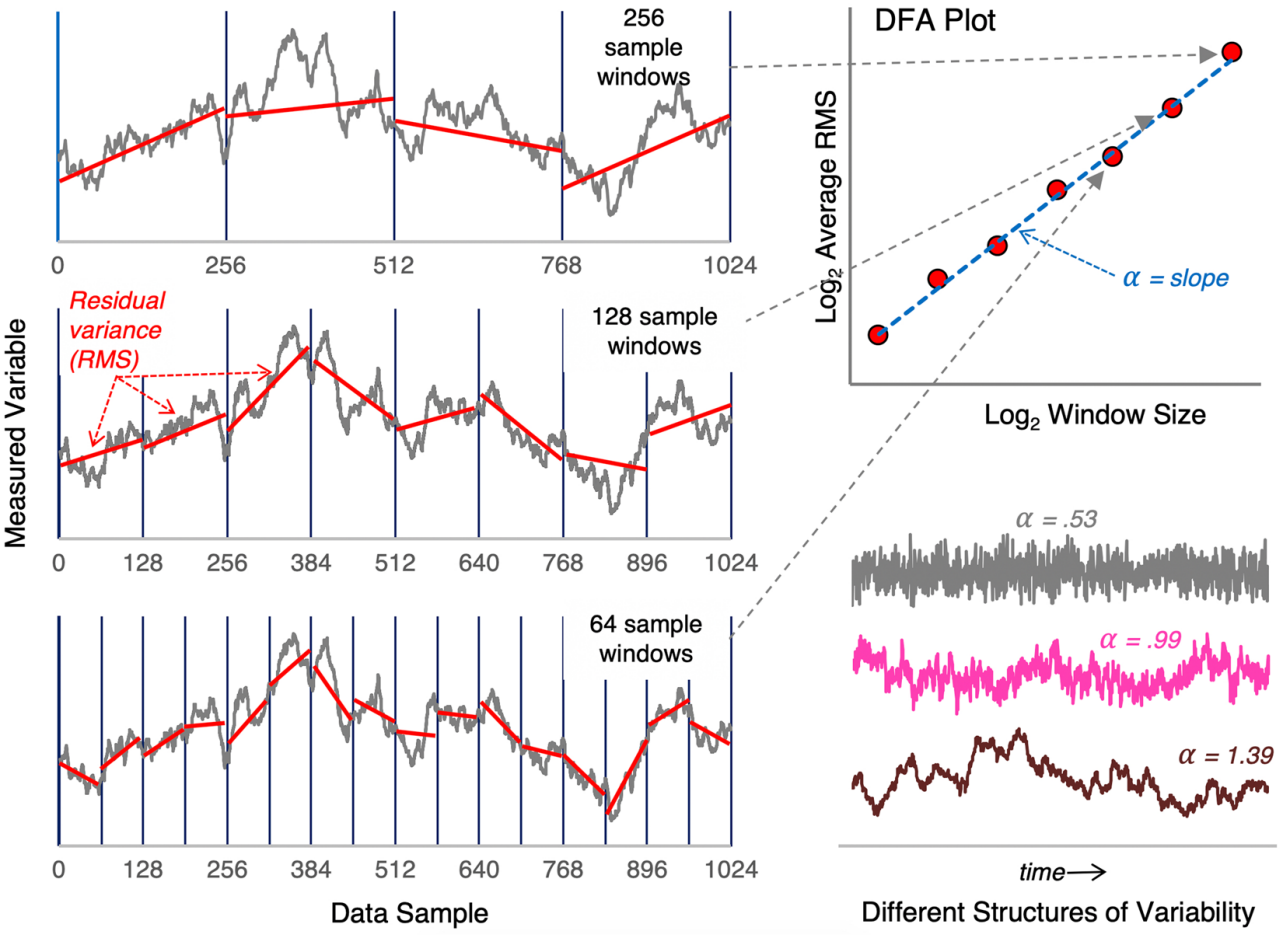
Illustration of *Detrended Fluctuation Analysis* (DFA) and example behavioral time series with different structures of variability. DFA determines the degree of random or persistent (deterministic) structure within a behavioral time series from the slope, *α* (alpha), of a log-log plot of the average residual variance (root-mean-square; RMS) as a function of the window size used to calculate the residual variance estimates. The bottom right time series are representative examples of *white* (random), *pink* (fractal, long-range correlated, slightly persistent), and *brown* (highly persistent) structures of variability.

There are a range of techniques that can be employed to determine the degree of self-similarity or “non-randomness” within a time series of behavioural fluctuations. One of the most commonly used methods is *Detrended Fluctuation Analysis* or DFA. As illustrated in Fig. 2, *DFA* determines the degree of self-similarity within a time series of behavioural fluctuations by calculating the average magnitude of variance across a range of window sizes (e.g., 8, 16, 32, etc. data points) and then plotting these estimates as a function of window size on a log-log plot. The slope of the regression line, *α*, that best fits the residual variance estimates in the DFA plot can then be employed as an index of self-similarity. In short, *α* ≈ 0.5 corresponds to a random, non-correlated, non-similar structure of variability (i.e., white noise); *α* ≈ 1 corresponds to self-similar, long-range correlated or persistent structures of variability (i.e., known as *fractal* or *pink noise*); and *α* ≈ 1.5 corresponds to highly persistent and correlated patterns of Brownian variability (i.e., so-called Brown noise), with most time series of human behavioural fluctuations typically resulting in 0.5 < *α* < 1.5^11,17,18^.

Importantly, the degree to which human behavioural variability is non-random or self-similar is assumed to reflect the complexity or nested interdependence of the sensorimotor, cognitive, social and environmental processes that define a given task context^11^. Consistent with this assumption, there is now a growing body of research demonstrating how the structure of behavioural fluctuations varies across different experimental manipulations and tasks constraints^10,12,14,19–22^. For instance, Dotov, Bardy, & Dalla Bella^19^ investigated the effects of environmental constraints on walking dynamics using the *α* exponent from participants’ time series of stride durations. They found that *α* decreased significantly when participants had to continuously steer along an elliptical pathway (constrained), versus when they were freely walking along an athletic track (unconstrained) and decreased further when participants had to walk within a rectangular area (and thus perform frequent 90 degree turns). Similarly, Chen *et al*.^23^ found that the structure of tapping variability was altered for tasks involving synchronization (more constrained) versus syncopation (less constrained) tapping to a metronome. They observed that the tapping variability in synchronization exhibited whiter structure (lower *α*) as compared to syncopation which exhibited pinker structure (higher *α*).

The relationship between constraints and the structure of variability has also been studied in the context of motor learning, under the hypothesis that when someone becomes highly skilled in a behaviour, the coordinative structure underlying the behaviour changes to better suit the demands of the task. For instance, Wijnants *et al*.^24^ utilized a precision aiming task methodology in which participants repeatedly drew a line back and forth between two visual targets. They observed that the variability in participant’s movement times between targets became increasingly fractal-like alongside the development of their expertise in aiming, suggesting that their motor learning resulted in changes in the flexible organization of coordinating subsystems.

More recently, Washburn *et al*.^14^ investigated the relationship between constraints and structure of variation by exploring the ways in which this structure changes in response to differing task intentions. In this experiment, participants attempted to either repeatedly swing their right forearm at a specific amplitude (spatial/movement control), or at a constant frequency (temporal control). The results revealed that the structure of variation for amplitude control and temporal control was only affected by the corresponding intentional state. For instance, having the intention to control movement tempo, resulted in whiter, more random variability for movement frequency, but had no effect on amplitude variability. The importance of these latter results is that they provide evidence that an actor’s task intention or goal can operate to constrain or change the dynamic structure of performance variability in a similar way as physical or informational constraints do.

### Social coordination and complexity matching

Many everyday tasks are performed within a social context. Research over the last four decades has demonstrated how the movements and actions of co-present or co-acting individuals often become entrained or coordinated, even when coordination is not required^25,26^. For instance, research has demonstrated how individuals often unintentionally mimic the actions, postures or movements of other conspecifics during social interaction^27–29^. Conversing individuals tend to become posturally entrained^30–32^, as well as exhibit coordinated eye-looking behaviour^33,34^, and even converge in their speaking rate, accent and word use^26,35–37^. Moreover, research on the dynamics of social motor coordination has demonstrated how individuals often synchronize their limb and body movements during co-present interaction and that in many instances, co-actors are unaware of their behavioural synchrony^38–40^. For instance, individuals sitting side by side in rocking chairs tend to synchronize their rocking movements^41^. Likewise, two or more individuals walking or running together tend to synchronize their steps and leg movements^42–44^. Research has also shown how such behavioural synchrony can have pro-social benefits, with instances of unintentional behavioural synchrony increasing rapport and reducing perceived self-other differences^45–49^.

Of more significance here, however, is recent research indicating that interacting individuals also tend to exhibit coordinated structures of behavioural variability. As noted above, this phenomenon is known as *complexity matching*^50–52^, and refers to the patterning of one individual’s behavioural variability closely matching or become aligned with that of other co-actors. Importantly, complexity matching does not require that the movements, actions or behaviours of interacting individuals be synchronized or spatiotemporally correlated at a local or short-term scale^53,54^. Instead, complexity matching can refer to a convergence of behaviour at a more global, non-local level, such that the behavioural fluctuations of co-actors begin to exhibit similar patterns or structures of variability (i.e., the same patterns of non-randomness or self-similarity) independent of any local coordination or synchrony. In this sense, the alignment observed during complexity matching indicates that the co-actors behavioural dynamics have become entrained to the overall statistical patterning of each other’s behavioural fluctuations^12^.

Evidence that complexity matching can occur during social interaction was first discovered in interpersonal rhythmic-limb coordination, where members of a dyad were found to exhibit similar (correlated) structures of variability independent of the stability of the rhythmic coordination observed^55^. Similarly, forms of complexity matching have been observed in interpersonal tapping tasks in which dyads are engaged in tapping with one another^56^. Complexity matching has even been shown in two participants’ stride durations while engaging in side-by-side walking and arm-in-arm walking, where the correlations between participants’ mono and multifractal spectra were shown to converge to a high degree for both forms of interpersonal walking^57^.

Complexity matching has also been shown to occur during more complex interactive behaviours. For example, a recent study utilized DFA to investigate complexity matching in the context of rowing^58^. They found that athletes engaging in side-by-side rowing were strongly and significantly correlated in their DFA *α* exponents, as compared to completely uncorrelated during individual sessions. Interestingly, they found that this complexity matching was not due to the athletes mimicking or locally adapting to each other, indicating a more global coordination pattern emerging between them. Additionally, Abney *et al*.^59^ found evidence for complexity matching in the conversational dynamics of two interacting individuals using the time series of acoustic onsets of their conversation. This complexity matching was only observed in affiliative conversations as opposed to argumentative conversations, indicating that this phenomenon is sensitive to changes in interpersonal context. More recently, Schnieder *et al*.^60^ observed complexity matching during bilingual and monolingual conversations in the hierarchical timing of speech and in the frequency distributions of lemmas. These two types of convergence were observed equally in English-only, Spanish-only, and bilingual English-Spanish conversations, suggesting that the occurrence of complexity matching is robust to monolingual vs bilingual interactions and, thus, may stem from more rudimentary processes involved in coordination and communication.

### Current study

As noted above, the majority of the research investigating complexity matching and the structure of human behavioural variability across different task contexts has been conducted using highly constrained, laboratory-based experimental tasks. Thus, it remains to been seen whether these same results can generalize to everyday, naturalistic task activities. This is particularly true with regards to complexity matching, as there is no previous research verifying the occurrence of complexity matching during naturalistic interpersonal interaction outside of a laboratory setting. Accordingly, the current study investigated (i) whether the structure of human behavioural variability is sensitive to changes in environmental and task constraints and (ii) whether the structure of two individuals’ behavioural variability become more similar/aligned (i.e., complexity matching occurs) during the completion of everyday task activities.

More specifically, we assessed the structure of the motion (acceleration) variability of individuals (individual condition) or pairs of individuals (pair condition) during the performance of five on-campus student activities: (1) walking through a campus stadium with stairs; (2) visiting the campus student centre, (3) performing a library search task; (4) visiting a campus garden; and (5) walking across campus from one location to another (see Table 1 for more details). These activities were chosen to reflect everyday student activities embedded in familiar, commonplace environments and, moreover, entail different levels of task/goal constraint. In short, the two walking activities involved a clearly defined task goal and entailed highly regular and cyclical movements and navigational actions, and thus represented more constrained task contexts compared to the two free-form activities, while the library search task entailed a combination of directed and exploratory actions. Thus, we expected to see a slightly whiter structure of variability in the walking tasks (i.e., *α* «1) as compared to the library and the two free form tasks, which were all expected to exhibit somewhat pinker (i.e., *α* closer to *1)* patterns of motion variability.

**Table 1:** Labels and summaries of all five activities used in analysis.

Activity Name	Activity Details
Stadium Walk	Participants walk through a football stadium, including sets of stadium stairs
Student Centre	Participants spend approx. 15 mins free-form in the student centre
Library Search	Participants search for several items throughout multiple floors of the campus library
Garden	Participants spend approx. 15 mins free-from in a campus garden
Campus Walk	Participants walk across campus (approx. 150 meters)

**Figure 3:**
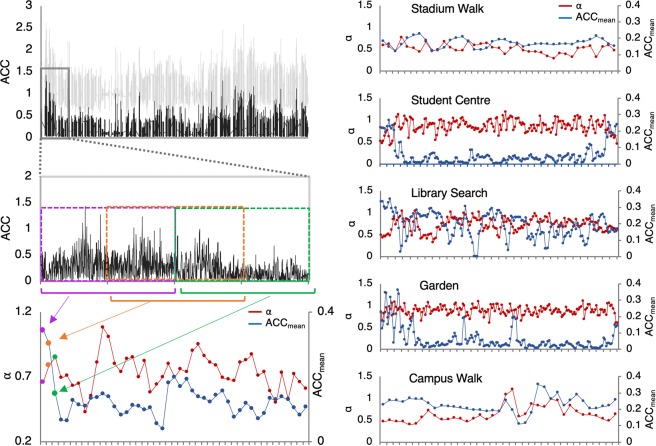
Illustration of the analysis process and example data. *Left column:* Illustration of the windowed (epoch) analysis process, wherein *α* and ACC_mean_ are extracted within sliding windows of 1024 points with 50% overlap, resulting in a time series for each of these measures. The top left graph displays the raw magnitude of accelerometer in light grey, and the filtered, centred and rectified magnitude of accelerometer in black. The middle left graph zooms into the first 2048 points from the top left graph. The purple, orange, and green boxes illustrate the first three sliding windows with 50% overlap: 1 s) 1:1024, 2) 512:1536, 3) 1024:2048 points. Within each of these windows, DFA is computed to extract *α*, and the average of the points is computed to extract ACC_mean_. The resulting time series for *α* and ACC_mean_ are shown in the bottom left graph, with the first three measures for *α* and ACC_mean_ coloured according to their respective window. *Right column:* Example time series of *α* and ACC_mean_ for the five activities.

The motion dynamics of individual and paired participants was recorded using a wearable wrist (Empatica E4) accelerometer. Note that waist motion was also recorded using waist-mounted iPhone and produced similar results; see Supplementary Material. The structure of the motion variability produced by individuals during the different task activities was determined using *windowed-DFA*, whereby *α* was calculated separately across sliding windows of 1024 points with a 50% overlap (illustrated in Fig. 3). This resulted in a time series of *α* values for each of the five activities (and for each participant) with average *α*, hereafter referred to as *α*_mean_, employed as an index of the overall structure of variability of the accelerometer data for a given activity.

The mean magnitude of acceleration was also calculated using a sliding window of 1024 points with 50% overlap. The average of the means from this magnitude of acceleration time series was then used as the overall linear measure of motion magnitude for the five activities, with the mean magnitude of acceleration hereafter referred to as ACC_mean_. Note that the same process was used to calculate a time series of the standard deviation of magnitude of acceleration. This measure is not reported here, however, as the results were wholly redundant with the results for ACC_mean_.

The degree of complexity matching that occurred within pairs was indexed by determining the degree to which the structure of variability of individuals in a pair covaried during the different activities. Traditionally, a correlation between the overall *α* values of co-acting individuals is used to calculate the degree of complexity matching between two systems^55^. For the current study, however, a cross correlation analysis was conducted using the two *α* time series belonging to each member in a pair (obtained via the windowed DFA analysis), with the resulting Fisher-z transform of the correlation at lag zero used to determine the association between the *α* values of paired individuals for each activity. Note that max correlation occurred at lag zero over 96% of the time for both *α* and ACC_mean_ time series.

A pseudo pairs analysis was also employed to determine the degree to which the co-variance in *α* (i.e., complexity matching) was a function of co-participant interaction and not simply due to performing the same tasks in the same environment. Two types of pseudo-pairs were created. The first set of pseudo-pairs was created from the actual pairs and corresponded to pairing all possible combinations of individuals in the pair condition that did not complete the experiment together (for a total of 684 pseudo-pairs). The second set of pseudo pairs were created from participants who completed the experiment alone. Again, all possible combinations of participants from the individual condition were paired together (for a total of 153 pseudo-pairs). Zero-padding was used when needed to ensure that time series within a pair were of equal length.

## Results

### Structure of behavioural variability

**Figure 4:**
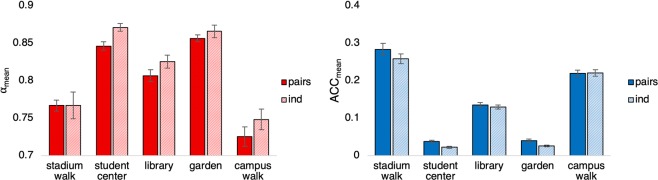
Mean dependent measures for wrist acceleration magnitude displayed for all five activities. Error bars represent standard error *Left:* Mean *α*, with pairs in dark red; individuals in light red. *Right:* Mean acceleration magnitude, with pairs in dark blue; individuals in light blue.

The means for both of the dependent measures (i.e., *α*_mean_ and ACC_mean_) as a function of activity and condition are displayed in Fig. 4. In order to confirm whether the five behavioural activities (stadium stairs, student centre, library search, garden, campus walk) differentially influenced the motion variability exhibited by participants, separate 2 (condition: individuals vs. pair) x 5 (activities) repeated measures ANOVAs were conducted for both of the dependent measures (i.e., *α*_mean_ and ACC_mean_). For pairs, each dependent measure was averaged across both participants in a pair, resulting in a single value for each dependent measure for each pair. Greenhouse-Geisser corrections were employed wherever the assumption of sphericity was violated (according to Mauchly’s test). Post hoc analyses (pairwise comparisons) were conducted using the Bonferroni correction.

As expected, the analysis of *α*_mean_ revealed a significant main effect of activity, *F*(2.27, 79.57) = 61.68, *p* < 0.001, η_p_^2^ = 0.638, with post hoc analyses revealing that the two walking activities resulted in significantly whiter (i.e., *α* values closer to 0.75) movement variability than the other three activities (all *p* < 0.05) and the two free form activities, i.e., student centre and garden, resulted in significantly pinker (i.e., *α*> 0.85) movement variability than the other three activities (*p* < 0.05). The library activity was characterized by *α*_mean_ values ≈ 0.825, and thus fell in-between the walking activities and free form activities (*p* < 0.05). The analysis of *α*_mean_ also resulted in a main effect of condition, with pairs having significantly lower overall *α*_mean_ as compared to participants who completed the activities alone, *F*(1, 35) = 5.56, *p* < 0.05, η_p_^2^ = 0.137, indicating that the presence of a co-actor may have operated as another constraint on the behavioural motion of individuals in the pair condition^61^. There was no interaction between activity and condition, *F*(2.27, 79.57) = 0.534, *p* = 0.611, η_p_^2^ = 0.015.

The analysis of ACC_mean_ also revealed a significant effect of activity, *F*(1.791, 62.67) = 398.7, *p* < 0.001, η_p_^2^ = 0.919, with post hoc analyses revealing that the two free form activities had significantly lower ACC_mean_ than the other three activities (all *p* < 0.05). The remaining three activities were all significantly different from one another (all *p* < 0.05). There was no main effect of condition, *F*(1, 35) = 3.2, *p* = 0.08, η_p_^2^ = 0.084, nor an interaction between activity and condition *F*(1.791, 62.67), *p* = 0.426, η_p_^2^ = 0.023.

### Complexity matching

**Figure 5:**
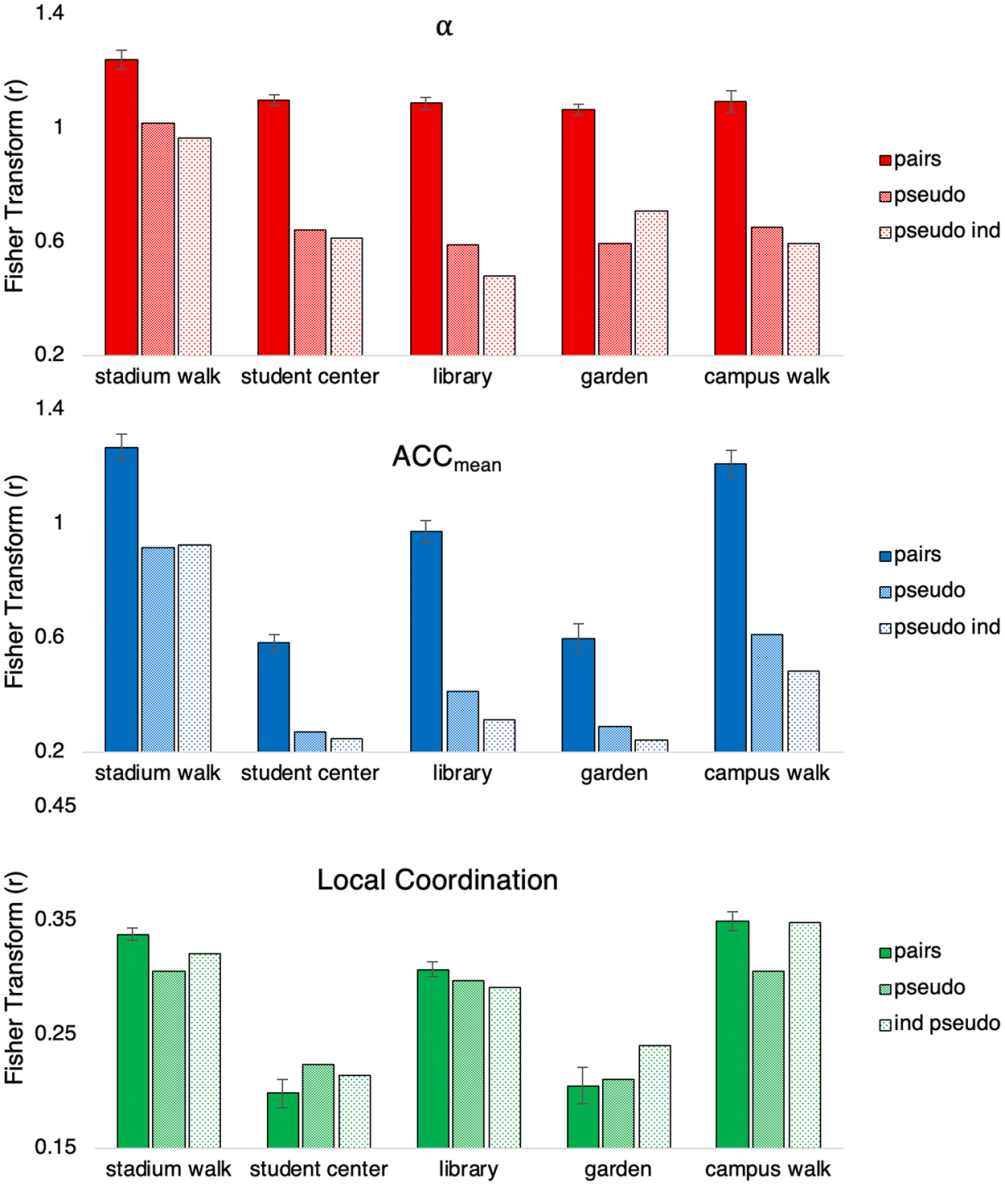
*Top:* Complexity matching results. Fisher’s transform of the cross correlation (*r)* between *α* values of wrist acceleration for members in a pair or pseudo pair. *Middle:* Fisher’s transform of the cross correlation (*r*) for ACC_mean_. *Bottom:* Local coordination results. Fisher’s transform of the cross correlation (*r*) between the acceleration magnitude time series. In all graphs, original pairs are shown in the darkest colour, pseudo pairs generated from pairs in mid-light colour, and pseudo pairs generated from solo individuals in the lightest colour. Error bars represent standard error.

Recall that the degree of complexity matching that occurred between members of a pair was assessed by calculating the cross-correlation between the co-actors’ time series of *α* values. As can be seen from an inspection of Fig. 5 (top), high levels of complexity matching were observed for all of the different activities.

In order to test whether the complexity matching seen for the actual pairs was also present in the pseudo pairs, separate one-sample t-tests were run to compare the set of cross-correlation values of the actual pairs to the mean cross-correlation values of the pseudo pairs. This analysis revealed that the cross-correlations observed for the actual pairs was significantly higher than the cross-correlations observed for both types of pseudo pairs (i.e., pseudo pairs made from the participants who completed the activities in a pair, and pseudo pairs made from the participants who completed the activities alone), and this was true for all five activities (all *t* > 6.7, *p* < 0.05).

To test whether the degree of complexity matching varied as a function of activity, separate one-way repeated measures ANOVAs were conducted on the Fisher-z transform of the cross-correlation coefficients. Interestingly, although this analysis revealed a main effect of activity, *F*(2.77, 49.82) = 7.06, *p* < 0.05, η_p_^2^ = 0.282, a post hoc analyses revealed that only the stadium walk activity exhibited a slightly higher degree of *α* covariance than the other four activities (all *p* < 0.05). These results indicate that the degree of complexity matching that occurs between co-actors is relatively invariant across task activity (as discussed in more detail below), and thus might provide a robust, task-independent indicator of co-action.

To check whether the complexity matching results were not simply due to changes in overall movement magnitude, a cross correlation analysis of ACC_mean_ was also conducted for the actual and pseudo pairs (also at lag zero and using Fisher-z transform). Again, separate independent samples t-tests were run to compare the overall mean cross-correlation values of the pseudo pairs to the set of cross-correlation values of the original pairs.

Similar to the *α* covariance results above, the degree of ACC_mean_ cross-correlation was also significantly higher for real pairs compared to both types of pseudo pairs, for all five activities (all *t* > 5.4, *p* < 0.05). However, a one-way repeated measures ANOVA examining the differences in the Fisher-z transformed ACC_mean_ cross-correlation coefficients (with Greenhouse-Geisser corrections employed wherever necessary), revealed a significant and rather large effect of activity, *F*(4, 72) = 90.48, *p* < 0.001, η_p_^2^ = 0.834, indicating that ACC_mean_ covariance was not invariant across task activity. Indeed, in contrast to *α* covariance (i.e., complexity matching), post hoc analyses (Bonferroni correction) revealed that the two walking activities exhibited much higher degrees of cross-correlation than the other three activities (all *p* < 0.05), with the two free form activities exhibiting much lower degrees of ACC_mean_ covariance than the other three activities (all *p* < 0.05); see Fig. 5 left middle.

To determine whether the complexity matching observed was simply due to local (synchronous) coordination processes, a cross-correlation analysis was also conducted on the motion time series of members of a pair or pseudo pair. This analysis indexed the degree to which the moment-to-moment movements of members of a pair were synchronized. This analysis exhibited weak-to-moderate degrees of local (synchronous) coordination (see Fig. 3 bottom row). However, one sample t-tests revealed that the correlations for the actual pairs were not significantly different from the correlations observed for one or both sets of pseudo pairs for all the activities, except the stadium walk (both *t* > 3.43, *p* < 0.05), implying that the complexity matching results reported above were not simply a result of local movement coordination or synchrony. More specifically, correlations observed for pairs were not significantly different from both sets of pseudo pairs for the student centre activity (both *t* > −1.24, *p* > 0.05). For the garden activity, the correlations for the actual pairs were not significantly different from the pseudo pairs made from the pairs (*t* = −0.35, *p* > 0.05), and were significantly lower than the pseudo pairs made from the solo individuals (*t* = −2.28, *p* < 0.05). For the library activity, the correlations for the actual pairs were also not significantly different from the pseudo pairs made from the pairs (*t* = 1.65, *p* > 0.05), but were significantly higher than the pseudo pairs made from the solo individuals (*t* = 2.48, *p* < 0.05). For the campus walk activity, the correlations for the actual pairs were not significantly higher than the pseudo pairs made from the solo individuals (*t* = 0.17, *p* > 0.05), but were significantly higher than the pseudo pairs made from the pairs (*t* = 5.22, *p* < 0.05).

Finally, the degree of local movement coordination exhibited between pairs also varied greatly across the various activities. A one-way repeated measures ANOVA on the Fisher-z transformed cross-correlations coefficients resulted in a significant effect of activity, *F*(2.51, 45.14) = 76.06, *p* < 0.001, η_p_^2^ = 0.809 (see Fig. 5, bottom). Indeed, in contrast to the relative invariance of the complexity matching results with respect to activity, post hoc analyses with Bonferroni correction revealed that co-actors exhibited significantly higher levels of local coordination during the two walking activities compared to the library, student centre and garden activities (all *p* < 0.01). Additionally, the two free-form activities exhibited significantly lower levels of local coordination than the remaining three activities (all *p* < 0.01).

## Discussion

The objectives of the current study were to investigate whether the structure of human behavioural fluctuations is co-related to changes in the structure of naturalistic activities, as well as whether co-acting pairs exhibited convergence in their structure of variability during the performance of everyday activities. Participants completed five on-campus activities, which included two walking activities, a goal-directed activity, and two free-form activities. The participants’ behavioural activity was recorded via accelerometer and GPS housed within wearable technology. An analysis of the *α* and ACC_mean_ time series of participants’ magnitude of acceleration at the wrist was conducted in order to address the study objectives.

As expected, the results of the DFA analysis revealed that the structure of the participants’ behavioural fluctuations (i.e., *α*_mean_) was indeed co-related to changes in the activities the participants performed. Specifically, the structure of variability at the wrist was overall whiter (i.e, closer to random variation) for the two walking activities and pinker (i.e., closer to fractal variation) for the two free-form activities, while the library search activity was in between the walking and free-form activities. These results could reflect the regularity and repetitiveness of the motion or movements produced during walking and, in contrast, the varied spontaneity of behaviour (and thus motion) during the free-form activities. These results support the idea that the structure of behavioural fluctuations varies with task or environmental constraints, which has only been demonstrated previously using various simple experimental motor tasks^14,23,24^. The results also reveal how participants’ mean magnitude of acceleration (i.e., ACC_mean_) was modulated by changes in activity and task goal. However, *α*_mean_ appeared to be more successful in grouping similar activities together (i.e, free-form and walking), while ACC_mean_ only grouped together the free-form activities. Furthermore, *α*_mean_ was able to differentiate between individuals who completed the activities alone, versus those who completed the activities in a pair.

With regards to complexity matching, both the cross correlations of *α* (complexity matching) and ACC_mean_ resulted in significantly higher values than the pseudo pairs. However, the degree of cross-correlation for ACC_mean_ varied significantly as a function of activity type while the degree of complexity matching did not appear to be sensitive to these changes, indicating that the co-variance of *α* values was a more stable and reliable method of indicating whether two individuals were behaving together regardless of their current activity. The cross-correlation measure of local coordination was also significantly modulated by activity. Moreover, the magnitude of local coordination between co-actors remained rather weak across all the different activities and, in many cases, was not significantly different from the chance (baseline) levels of local coordination determined from the pseudo pairs analysis. Thus, not only did the co-variance of *α* provide the most robust, task-independent indicator of co-action, it did so somewhat independent of the local coordination process that may have occurred between co-actors. This stability in the co-variance of *α* across the activity types therefore implies that complexity matching could be used as a task general method for indexing coordinated social interaction.

The use of wearable technology in this experiment allowed for participants’ behavioural activity to be collected relatively unobtrusively within natural environmental settings in order to help address some of the methodological pitfalls of previous research, which has primarily relied on artificial laboratory settings^62^. However, the nature of the data collected in this experiment is both a strength and a pitfall, as naturalistic data can be messy and uncertain. Within the same activity, participants reported engaging in a range of behaviours. For example, the student centre activity involved some combination of sitting, walking, conversing and internet browsing. This range of behaviours is a true reflection of the behavioural variability found within the same environmental space, but it also negates any possibility of highly specific hypotheses and conclusions. As such, future research into naturalistic behaviour would benefit greatly from some form of unbiased monitoring, such as wearable cameras, to capture more specific activity labels^63^.

Finally, in addition to validating the occurrence of complexity matching during naturalistic interaction, the current results also have potentially significant implications for machine-based social activity recognition. There is growing interest in developing algorithms or neural networks that are capable of discerning whether two people are interacting/co-acting versus just independently behaving within the same environmental space, and much of the current research utilizes surveillance video footage and/or a combination of sensor data^64–67^. The current results show that by simply using the structure of variability of accelerometer magnitude, one could employ complexity matching analysis to detect when two people are engaged in an activity as a pair, versus engaged in that same activity alone. This also suggests that interacting individuals are not only coupled to the structure of the environment and the task, but also coupled to each other in such a way that they form a unified social system. Thus, future research could continue to explore how this method can be utilised for robust detection of interacting/co-acting individuals within various activity types and environmental contexts.

## Method

### Participants

78 undergraduate students (26 individuals and 26 pairs) from the University of Cincinnati participated in the study for course credit. Eight individuals and seven pairs were eliminated due to either equipment failure or failure to complete the task activities. The 56 remaining participants (18 individuals and 19 pairs) ranged from 18 to 39 years old (*M* = 19.2, *SD* = 2.9) with 45% of the participants being male. This study was approved through the University of Cincinnati Institutional Review Board (IRB Approval Code: 2012–2827). Informed consent was obtained from all participants. All methods were performed in accordance with the relevant guidelines and regulations.

### Equipment

Acceleration at the non-dominant wrist was recorded using an Empatica E4 wristband (Empatica Inc., Milano, MI), which is equipped with a 3-axis accelerometer (range [−2g, 2 g]) sampled at 32 Hz. The participants GPS location was recorded continuously during the experiment using the open-source CrowdSense App^68^ on an iPhone SE (Apple Inc, Cupertino, CA). GPS data was updated at approximately 1 Hz and was employed to identify when the different activities began and ended (see section ‘Activity Detection’ for more details).

### Design and procedure

The Empatica E4 wristband was placed on the participant’s non-dominant wrist and the iPhone SE was clipped to a holster on the waist. All participants were instructed to keep the iPhone clipped to the holster and the Empatica E4 wristbands on their wrists for the duration of the experiment. Participant dyads were told to stay together for the entire experiment.

Note that acceleration at the waist was also recorded using the iPhone SE. However, these results were quite similar to the results for the wrist, and thus, for simplicity, only results for wrist acceleration are shown in this paper. All results for acceleration at the waist are included in the Supplementary Material.

Participants were instructed to perform a series of five simple activities (either alone or in dyads) that required them to take similar paths around the University of Cincinnati (UC) campus. Participants were given a piece of paper with these instructions in full detail to take with them throughout the experiment, and all participants completed the tasks in the same order. Note that the experiment also included a sixth activity in which participants visited an on-campus coffee shop. This activity was removed from all analyses, however, due to the high degree of variability in the context and time spent in the activity region (ranging from approximately 2–20 minutes depending on the queue length).

### Data Pre-processing

The magnitude of acceleration at the wrist from the Empatica E4 wristband was obtained for analysis. The XYZ wrist acceleration data from the Empatica E4 wristband was used to calculate a vector magnitude of acceleration at the wrist as a single time series, which was then centred, rectified and filtered using a 4^th^ order 10 Hz low-pass Butterworth filter.

### Activity detection

The final pre-processing stage involved partitioning the full time series into separate activity time series for each of the five activities: (1) stadium walking, (2) (free-form) student centre, (3) library search, (4) (free-form) garden, (5) campus walking. To this end, an algorithm was developed using Python 3.6 to detect the timestamps at which a participant entered and exited a circular radius around the approximate centre point of each activity location using the iPhone SE GPS data. The timestamps associated with these points of entry and exit were then used to slice the magnitude of acceleration time series for the wrist into separate windows corresponding to each activity. The algorithm utilized Vincenty’s (1975) formulae (inverse method) to compute the azimuth and geographical distance between two given geo-points.

Note that for the stadium walking activity, due to the high variability in the entry and exit positions across participants, the algorithm was used to detect only the start time of the activity (timestamp at which they entered the stadium), while the end of this window was set to 3.5 minutes after the start time. The algorithm was used for detecting both the start and end times for all of the other activities. In order to validate the accuracy of the resultant windows, GMapPlot from Bokeh, an open-source Python library, was used to overlay the GPS data for each activity on a satellite image of the University of Cincinnati campus through Google maps, which allowed for visual validation of each activity time series.

## Supplementary information

Supplementary Materials.

## Data Availability

The datasets generated and analysed during the current study are available in the Open Science Framework (OSF) repository: https://osf.io/z47qw/?view_only=53c29a229d7940c38bf288b72e8471f2.
