# Supplementary material for: Co-actors Exhibit Similarity in Their Structure of Behavioural Variation That Remains Stable Across Range of Naturalistic Activities
Source: Sci Rep. 2020 Apr 14;10:6308. doi: 10.1038/s41598-020-63056-x (PMC7156677; doi:10.1038/s41598-020-63056-x)
Supplement: Supplementary file 1 — Supplementary Materials. [file 41598_2020_63056_MOESM1_ESM.docx]

Supplementary Materials

Co-actors Exhibit Similarity in Their Structure of Behavioural Variation That Remains Stable Across Range of Naturalistic Activities

^1,2^Lillian M. Rigoli*, ^2,3,4^Tamara Lorenz, ^5^Charles Coey, ^1,6^Rachel Kallen, ^7^Scott Jordan and ^1,6^Michael J. Richardson*

^1^ Department of Psychology, Macquarie University, Sydney, New South Wales, Australia

^2^ Center for Cognition, Action & Perception, Department of Psychology, University of Cincinnati, Cincinnati, OH, USA

^3^ Department of Mechanical and Materials Engineering, University of Cincinnati, Cincinnati, OH, USA

^4^ Department of Electrical Engineering, University of Cincinnati, Cincinnati, OH, USA

^5^ Osher Center for Integrative Medicine, Harvard Medical School and Brigham and Women’s Hospital, Boston, MA.

^6^ Centre for Elite Performance, Expertise and Training, Macquarie University, Sydney, New South Wales, Australia

^7^ Department of Psychology, University of Illinois, IL, USA

Structure of Behavioural Variability for Waist Magnitude of Acceleration

In order to confirm whether the five behavioural activities (stadium stairs, student centre, library search, garden, campus walk) differentially influenced the waist movement dynamics exhibited by individuals and pairs, separate 2 (condition: individuals vs. pair) x 5 (activities) repeated measures ANOVAs were conducted for both of the dependent measures (i.e., α_mean_, ACC_mean_). The dependent measures were averaged across both participants in a pair, resulting in a single value for each of the dependent measures for each pair. Greenhouse-Geisser corrections were employed wherever the assumption of sphericity was violated (according to Mauchly’s test). Post hoc analyses (pairwise comparisons) were conducted using the Bonferroni correction.

*Waist Acceleration*

For ACC_mean_, the analysis revealed a significant effect of activity, *F*(2.18, 76.19) = 417.74, *p* < .01, η_p_*²* = .923, but no significant interaction between activity and group, *F*(2.18, 76.19) = .094, *p* = .92, η_p_*²* = .003, and no significant main effect of group, *F*(1, 35) = .788, *p* = .38, η_p_*²* = .022. Post hoc analyses revealed that the two free form activities (student centre and garden) had significantly lower ACC_mean_ than the other three activities (all *p* < .05). The remaining three activities (stadium walk, library search, and campus walk) were all significantly different from one another (all *p* < .05).

For α_mean_, the analysis revealed a significant effect of activity, *F*(2.39, 102.4) = 261.8, *p* < .001, η_p_² = .882, but no significant interaction between activity and group *F(*2.39, 102.4) = 2.66, *p* =.053, η_p_*²* = .071. There was no significant main effect of group, *F*(1, 35) = .120, *p* = .73, η_p_*²* = .003. Post hoc analyses revealed that the two free form activities (i.e., student centre and garden) had significantly pinker movement variability than the other three activities (all *p* < .05). The remaining three activities (stadium walk, library search, and campus walk) were all significantly different from one another (all *p* < .05).


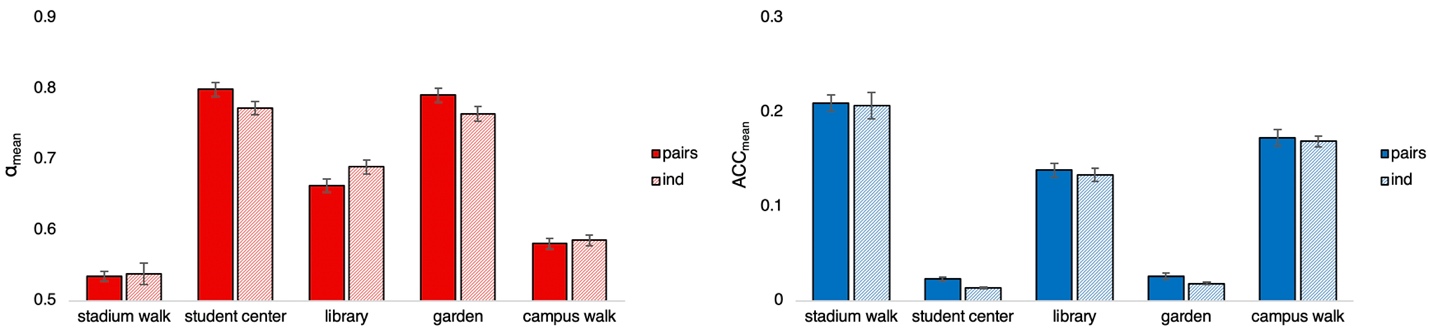


***Figure 1.*** Mean α (left; pairs in dark red; individuals in light red) and mean acceleration magnitude (right; pairs in dark blue; pairs in light blue) for waist acceleration magnitude displayed for all five activities. Error bars represent standard error.

Complexity Matching

In order to test whether the degree of complexity matching varied as a function of activity, separate one-way repeated measures ANOVAs were conducted on the Fisher Transform of the cross-correlation coefficient *r* for waist acceleration.

For the waist, the analysis revealed no main effect of activity, *F*(1.96, 35.34) = 1.67, *p* = .203, η_p_² = .085, indicating that the degree of cross-correlation remained stationary across all the different activities; see Figure 5.

The same cross-correlation analyses were repeated for the timeseries of the linear measure, ACC_mean_, to compare the complexity matching results with a simple cross-correlation of the linear movement magnitude measure. Again, separate independent samples t-tests were run to compare the overall mean cross-correlation values of the pseudo pairs to the set of cross-correlation values of the original pairs. This analysis revealed that the cross-correlations of the linear measures observed for the actual pairs were also significantly higher than the cross-correlations observed for both types of pseudo pairs, and this was true for both wrist and waist and for all five activities (all *t* > 5.4, *p* < .05).

In order to test whether the degree of cross-correlation varied as a function of activity, separate one-way repeated measures ANOVAs were employed to examine the differences in the Fisher Transform of *r* for waist acceleration. For ACC_mean_ of waist acceleration, the analysis revealed a significant main effect of activity, *F*(2.42, 43.49) = 13.07, *p* < .001, η_p_² = .421, with post hoc analyses revealing that the two walking activities (stadium walking and campus walking) exhibited significantly stronger degrees of cross correlation than the other three activities (all *p* < .05); see Figure 5.

Finally, the local, cross-correlation analysis conducted on of the full filtered time-series (measures taken from 0 lag) did reveal weak-to-moderate degrees of local (synchronous) coordination; see Figure 5. For the waist, one sample t-tests revealed that the correlations for the actual pairs was not significantly different than both sets of pseudo pairs for the stadium walk activity (both *t* < 2.06, *p* > .05). For the student centre, library, garden and campus walk activities, the correlations for the actual pairs was significantly higher than both sets of pseudo pairs (all *t* > 2.1, *p* < .05).

In order to test whether the degree of cross correlation varied as a function of activity, one-way repeated measures ANOVAs were again conducted on the Fisher Transform of *r* for the waist. For the cross correlation of waist magnitude of acceleration, the analysis revealed a main effect of activity, *F*(2.16, 38.8) = 28.17, *p* < .001, *η_p_²* = .61, with post hoc analyses revealing that the only the two free-form activities were not significantly different from each other (*p* > .05); see Figure 5.


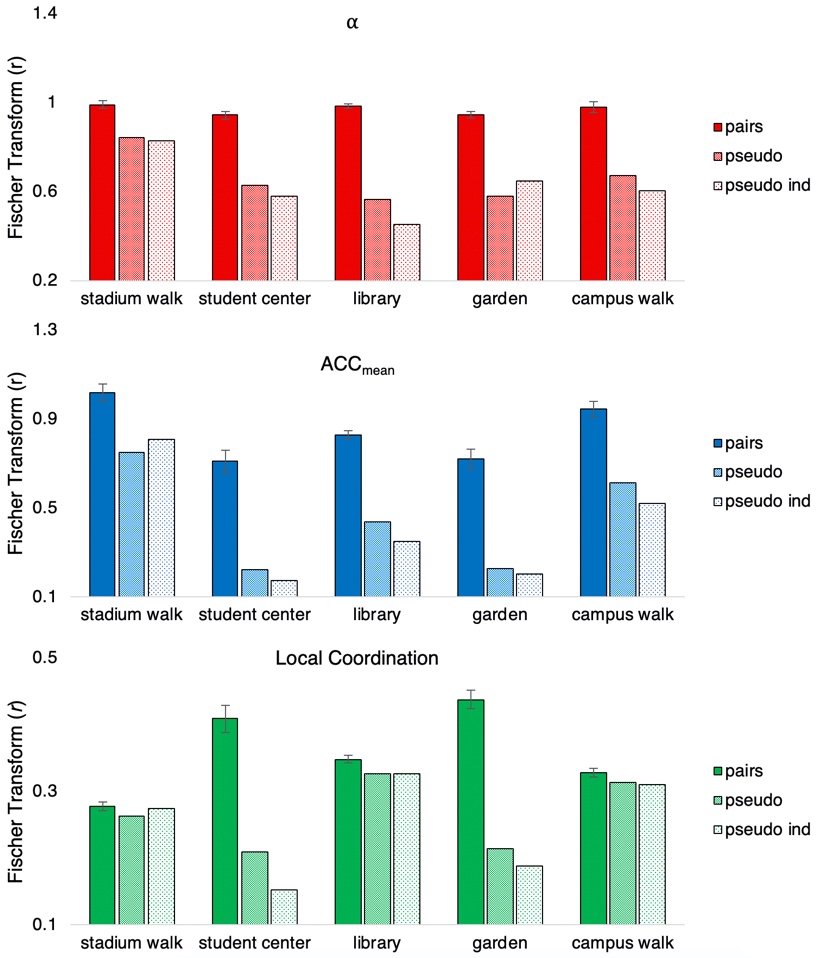


***Figure 2.*** *Top Row:* Complexity matching results. Fisher’s transform of the cross correlation (*r)* between α values of waist acceleration for members in a pair or pseudo pair. *Middle Row:* Fisher’s transform of the cross correlation (*r*) for ACC_mean_. *Bottom Row:* Local coordination results. Fisher’s transform of the cross correlation (*r*) between the acceleration magnitude time-series. In all graphs, original pairs are shown in the darkest colour, pseudo pairs generated from pairs in mid-light colour, and pseudo pairs generated from solo individuals in lightest colour. Error bars represent standard error.
